# Risk Factors for Carbapenem-resistant Enterobacterales Colonization Among Intensive Care Unit Patients in Korea

**DOI:** 10.1017/ash.2025.355

**Published:** 2025-09-24

**Authors:** GyeongJu Heo, JaHyun Kang

**Affiliations:** 1 Seoul National University Hospital; 2 Seoul National University College of Nursing

## Abstract

**Background:** Since the intensive care unit (ICU) is a high-risk area for healthcare-associated infections, effective infection control in the ICU is crucial. Carbapenem-resistant Enterobacterales (CRE) infections have been increasing and have become a significant concern for ICU patients. While CRE colonization does not require treatment, as it represents a carrier state, early detection is crucial to minimize CRE transmission. This study aims to identify the CRE colonization rate and its risk factors in ICU patients to provide a basis for CRE infection control in the ICU. **Method:** This retrospective cohort study was conducted at a university hospital in Korea from July 2023 to December 2023. Adult patients aged ≥18 years who were admitted to five ICUs (i.e., surgical 1 and 2, medical, cardiopulmonary, and emergency ICUs) and underwent active surveillance cultures for CRE using rectal swabs within 2 days of admission were included. Re-admissions and patients with confirmed CRE prior to admission were excluded. General, clinical, and environmental factor data were retrospectively collected using the hospital’s electronic medical records and nursing documentation system. Multivariate logistic regression was performed on variables with p **Results:** Out of a total of 1,473 ICU admissions, excluding duplicate admissions, 10 patients with confirmed CRE colonization prior to ICU admission and 722 patients who did not undergo active surveillance cultures within two days of admission were excluded. Among the remaining 741 included patients, 25 (3.37%) patients were colonized with CRE. Klebsiella pneumoniae was the most frequent isolate (n=18, 72%) and 12 patients (48%) were identified as having carbapenemase-producing Enterobacterales. In the multivariate logistic regression analysis, the following were identified as independent risk factors for CRE colonization: age (odds ratio [OR], 1.06; 95% confidence interval [CI], 1.01-1.11; p=0.036), admission from other hospitals (OR, 8.77; 95% CI, 2.43-31.59; p **Conclusion:** Based on the results of this study, early detection of patients with CRE colonization, followed by target screening and proactive infection control measures such as preemptive isolation, could play a key role in preventing the spread of CRE in the ICU.

